# Clinical Characteristics of 582 Patients with Uveal Melanoma in China

**DOI:** 10.1371/journal.pone.0144562

**Published:** 2015-12-08

**Authors:** Yue Ming Liu, Yang Li, Wen Bin Wei, Xiaolin Xu, Jost B. Jonas

**Affiliations:** 1 Beijing Tongren Eye Center, Beijing Tongren Hospital, Beijing Ophthalmology and Visual Science Key Lab, Capital Medical University, Beijing, China; 2 Beijing Institute of Ophthalmology, Beijing Tongren Hospital, Beijing Ophthalmology and Visual Science Key Lab, Capital Medical University, Beijing, China; 3 Department of Ophthalmology, Medical Faculty Mannheim of the Ruprecht-Karls-University, Heidelberg, Germany; University of Alabama at Birmingham, UNITED STATES

## Abstract

**Objective:**

To assess clinical characteristics, treatment and survival of patients with uveal melanoma in China.

**Methods:**

The retrospective study included all patients with malignant uveal melanoma who were consecutively examined in the study period from January 2005 and June 2015 in the Beijing Tongren hospital.

**Results:**

The mean age of the 582 patients (295(50.7%) women) was 44.6±12.6 years (range:5–77 years). The tumors were located most often in the superior temporal region (in 117(21.5%) patients) and least common in the inferior region (in 31(5.7%) patients). In 548(94.2%) patients, the tumors were located in the choroid, in 33(5.7%) patients in the ciliary body, and in one (0.2%) patient in the iris. Treatment included episcleral brachytherapy (415(71.3%) patients), local tumor resection (48(8.2%) patients) and primary enucleation (119(20.4%) patients). In 53 individuals out of the 415 patients with primary brachytherapy, episcleral brachytherapy was followed by enucleation, due to an increasing tumor size or due to uncontrolled neovascular glaucoma. Median follow-up time was of 30 months (range: 1–124 months; mean: 34.8 ± 24.4 months). Overall survival rate at 5 and 10 years was of 92.7% and 85.1%. Younger age (*P* = 0.017), tumor location in the nasal meridian(*P* = 0.004), smaller tumor size (*P*<0.001), hemispheric tumor shape (*P* = 0.025), histological tumor cell type (spindle-cell type versus epitheloid cell type;*P* = 0.014), and type of treatment (episcleral brachytherapy versus local tumor resection and versus primary enucleation; *P*<0.001) were significantly associated with the overall survival in univariate analysis, while in multivariate analysis only smaller tumor size was significantly (*P*<0.001; RR: 4.75; 95% confidence interval:2.11,10.7) associated with better overall survival.

**Conclusions:**

In this study on clinical characteristics of uveal melanoma of a larger group of patients from China, the onset age was considerably younger and survival rate better than in studies from Western countries. Tumor size was the most significant factor for survival.

## Introduction

Uveal melanoma is the most frequent primary malignant intraocular tumor in adults with an estimated incidence of 4–5 patients per million inhabitants and year in the United States and of 5–7.4 cases in European studies [1–4]. In a previous systematic review of existing databases, Singh and colleagues assessed the data of 4070 patients derived from the Surveillance, Epidemiology, and End Results program database in the United States from 1973 to 2008. These patients with uveal malignant melanoma representing 3.1% of all recorded patients with malignant melanoma showed an over-proportionally high percentage of white patients (97.8%) among the total group of patients with uveal malignant melanoma, and a higher age-adjusted incidence among men (incidence: 5.8 (95% confidence interval (CI): 5.5–6.2) than among women (incidence: 4.4 (95% CI: 4.2–4.7). In the study period from 1973 to 2008, the 5-year relative survival rate of approximately 81.6% remained mostly unchanged despite changes in the primary treatment, with a decline in the therapy option of surgery alone (93.8% for 1973–1975 versus 28.3% for 2006–2008) and a corresponding increase in the therapy option of radiation (1.8% for 1973–1975 vs. 62.5% for 2006–2008).

Although uveal malignant melanoma is the most common primary intraocular malignancy and despite the large population of China, there has been a scarcity of studies on patients with uveal melanoma from China. We therefore conducted this study to analyze the clinical characteristics, types of treatment and the survival rate of patients diagnosed with uveal melanoma within a period of 10 years, and to compare the results with those published from other populations.

## Methods

The retrospective hospital-based study included all patients who were consecutively diagnosed with primary uveal melanoma in the study period from January 2005 to June 2015 in the Beijing Tongren Hospital by the same surgeon (WBW). The study was approved by the Medical Ethics Committee of the Beijing Tongren Hospital, followed the tenets of the Declaration of Helsinki. Informed written consent was obtained from every study participant. All patients underwent a complete ocular examination including measurement of best corrected visual acuity, anterior biomicroscopy, tonometry, ophthalmoscopy, fluorescein angiography, and echography for measurement of the tumor dimensions. At six month intervals during the first two years and subsequently once per year, patients who received treatment of the uveal malignant melanoma additionally underwent sonography of the liver and biochemical blood analysis for liver-related enzymes. Imaging techniques additionally applied included magnetic resonance imaging, abdominal sonography and X-ray examination. Some patients also underwent abdominal computer tomography and/or positron emission tomography.

The classification based on size of uveal malignant melanoma was based on the criteria published by the Collaborative Ocular Melanoma Study (COMS) [5]. The total group was stratified by tumor size as measured by sonography. Tumors with a maximum height of 2.5 mm and a maximum base of 16 mm were classified as small tumors; medium-sized tumors had a height between 2.5 mm and 10 mm and a maximum base of 16 mm; and for large tumors, the maximal height was higher than 10 mm or the maximum base exceeding 16 mm [5].

All patients were treated by the same surgeon (WBW). The type of therapy depended on the size and characteristics of the tumors. Small tumors which did not show signs of activity or tumors of patients who rejected treatment were observed without intervention. Brachytherapy using I^125^ was applied for medium-sized tumors or for small tumor with documented tumor growth. Brachytherapy was also indicated for large tumors in which the patient rejected enucleation. Enucleation was performed for large tumors, if it appeared unlikely to recover visual function, for tumors with optic nerve invasion or extraocular extension, for tumors with a flat large infiltration of the choroid, or when the patient refused brachytherapy. The patients treated with brachytherapy and enucleation were re-examined at one month and three months after surgery, and after that once per half year. All enucleated globes were examined by the same pathologist. Parameters assessed were tumor cell type, basal tumor diameter and height, tumor location and position and tumor shape. Results of the histo-pathological examinations were available for those patients who underwent local resection or enucleation.

We used a statistical analysis program (SPSS 22.0; IBM-SPSS Inc., Chicago, IL, USA) for the statistical analysis of the data. The Kaplan-Meier method was applied to assess survival curves and the log-rank test was used for the univariate analysis of associations. The survival time was defined as the interval (measured in months) between the date of the initial therapy or supportive care and the date of death or the date of the last follow-up. Parameters which were significantly associated with the survival rate in the univariate analysis were included in the multivariate survival analysis using the Cox proportional hazards model. A *P*-value smaller than 0.05 was considered to be statistically significant.

## Results

The study included 582 patients (295 (50.7%) women; 293 right eyes, 289 left eyes) with a mean age of 44.6 ± 12.6 years (median: 44 years; range: 5 years to 77 years). Visual acuity in the affected eye was equal to or better than 0.80 in 70 (16.9%) patients, ranged between 0.50 and less than 0.80 in 66 (15.9%) patients, was between 0.10 and less than 0.50 in 165 (39.8%) patients, and it was lower than 0.10 in 114 (27.5%) patients. For 167 patients, visual acuity measurements were not reliable. Intraocular pressure was within the normal range of 10 to 21 mm Hg in 357 (86.9%) patients, while it was lower than 10 mmHg in 49 (11.9%) patients and higher than 21 mm Hg in 5 (1.2%) patients. For 171 patients, intraocular pressure measurements were not reliable. A retinal detachment was detected in 245 (71.6%) patients at the time of the diagnosis of the uveal melanoma.

The tumors were most often located in the superior temporal region (in 117 (21.5%) patients), followed by the temporal region (111 (20.4%) patients), the inferior temporal region (98 (18.0%) patients), the nasal region (62 (11.4%) patients), the superior nasal region (46 (8.4%) patients), the inferior nasal region (36 (6.6%) patients), the superior region (35 (6.4%) patients), and finally the inferior region (in 31 (5.7%) patients). In 9 (1.7%) patients, the tumor had extended over several regions. For 37 patients, the information on the location of the tumor was not sufficiently descriptive. In 548 (94.2%) patients, the tumors were located in the choroid, in 33 (5.7%) patients in the ciliary body, and in one (0.2%) patient the tumor was located in the iris. An optic disc involvement was detected in 13 (2.2%) patients.

Using the criteria of the COMS, there were 6 (1.1%) small tumors, 416 (77.9%) medium-sized tumors, and 112 (21.0%) large tumors. For 48 patients, the information on tumor size was not clear enough to allow a classification. With respect to the tumor form, the tumors had a mushroom-like shape in 117 (27.9%) patients, a mostly flat configuration in 11 (2.6%) patients, a hemispheric shape in 256 (61.1%) patients, and an irregular form in 35 (8.4%) patients. For 163 patients, the tumor shape was not sufficiently described to allow a clear allocation to a group.

Among the 159 patients for whom a histologic examination of a tissue specimen was possible and performed, 114 (71.7%) patients showed a spindle cell-type tumor, 17 (10.7%) patients had an epitheloid cell-type tumor, and 28 (17.6%) patients exhibited a mixed cell type tumor.

Treatment included episcleral brachytherapy in 415 (71.3%) patients, local tumor resection in 48 (8.2%) patients and primary enucleation in 119 (20.4%) patients. In 53 individuals out of the 415 patients with primary brachytherapy, episcleral brachytherapy was followed by enucleation, due to an increasing tumor size or due to uncontrolled neovascular glaucoma.

The median follow-up time was of 30 months (range: 1–124 months; mean: 34.8 ± 24.4 months). Follow-up was less than one year for 103 (17.7%) patients, more than one year and equal to or less than 3 years for 254 (43.6%) patients, more than three years and equal to or less than 5 years for 126 (21.6%) patients, more than five years and equal to or less than 10 years for 98 (16.8%) patients, and longer than 10 years for one (0.2%) patient.

Within the study period from January 2005 to June 2015, 32 patients (5.5%) died, among them 29 patients died due to a melanoma-related metastasis; one patient developed a second tumor, and two patients died due to pulmonary embolism and myocardial infarction (Table 1). A metastasis as examined in a general examination was detected in 17 (2.9%) patients, and 469 (80.6%) patients were alive without any detected metastases or tumor recurrences at the end of the follow-up period. For 64 (11.0%) patients, no follow-up data were available.

**Table 1:** Clinical characteristics of 582 patients with uveal melanoma.

Characteristics	Number of Patients	%
Age (Years) (Median: 44; Range: 5–77)		
0–20	13	2.2
21–40	203	34.9
41–60	301	51.7
61–80	65	11.2
Men / Women	287 / 295	49.3 / 50.7
Right / Left Eye	293 / 289	50.3 / 49.7
Visual Acuity (Measurements available for 415 Individuals)		
<0.1	114	27.5
0.1–0.49	165	39.8
0.5–079	66	15.9
≥0.8	70	16.9
Intraocular Pressure (mm Hg) (Measurements available for 411 Individuals)		
<10mmHg	49	11.9
10–21	357	86.9
>21mmHg	5	1.2
Retinal detachment	245	71.6
Position		
Superior	35	6.4
Nasal	62	11.4
Inferior	31	5.7
Temporal	111	20.4
Superior Temporal	117	21.5
Superior Nasal	46	8.4
Inferior Nasal	36	6.6
Inferior Temporal	98	18.0
Diffuse	9	1.7
Undetermined	37	
Location		
Ciliary Body	33	5.7
Iris	1	.2
Choroid	548	94.2
Optic Disk Involved	13	2.2
Size		
Small	6	1.1
Medium	416	77.9
Large	112	21.0
Undetermined	48	
Shape		
Mushroom	117	27.9
Flat	11	2.6
Hemisphere	256	61.1
Irregular	35	8.4
Undetermined	163	
Pathology (Available for 159 Individuals)		
Spindle-Cell	114	71.7
Epitheloid	17	10.7
Mixed	28	17.6
Initial Treatment		
Episcleral Brachytherapy (EB)	415	71.3
Local Resection	48	8.2
EB + Enucleation	119	20.4
Treatment		
Episcleral Brachytherapy (EB)	362	62.2
Local Resection	48	8.2
Primary Enucleation	119	20.4
EB + Enucleation	53	9.1
Outcome		
Live and No Metastasis	469	80.6
Death	32	5.5
Metastasis	17	2.9
Missing	64	11.0
Death reason		
Metastasis	29	90.6
Secondary Tumor	1	3.1
Pulmonary Embolism and Myocardial Infarction	2	6.3
Follow-Up Time (Years)		
≤1	103	17.7
>1 and ≤3	254	43.6
>3 and ≤5	126	21.6
>5 and ≤10	98	16.8
>10	1	.2

**Fig 1:**
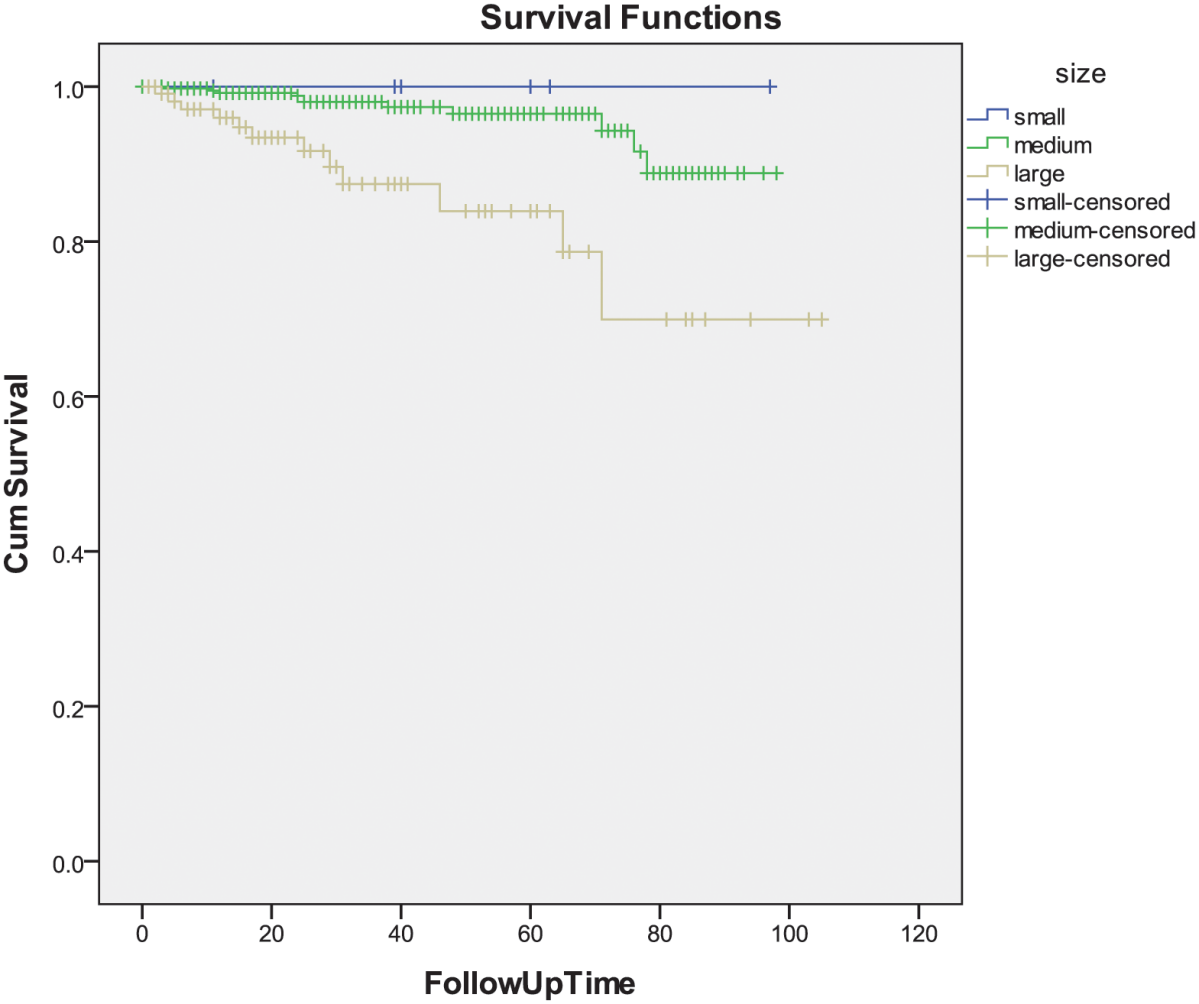
Graph Showing the Cumulative Survival (Cum Survival) in the Study Population.

Overall survival rate at 5 and 10 years was of 92.7% and 85.1%. Log-rank test analysis showed that younger age (*P* = 0.017), location of the tumor in the nasal meridian (*P* = 0.004), smaller tumor size (*P*<0.001), hemispheric tumor shape (*P* = 0.025), histological tumor cell type (spindle-cell type versus mixed cell type versus epitheloid cell type) (*P* = 0.014), and type of treatment (episcleral brachytherapy versus local tumor resection and versus primary enucleation(*P*<0.001) were significantly associated with higher overall survival. Survival was not significantly associated with gender (*P* = 0.55), right eye or left eye (*P* = 0.58), visual acuity (*P* = 0.50), intraocular pressure (*P* = 0.44), retinal detachment (*P* = 0.62), tumor location in the choroid or in the ciliary body (*P* = 0.90), and optic nerve head involvement (*P* = 0.37) (Table 2) (Fig 1).

**Table 2:** Univariate Survival Analysis by Kaplan-Meier.

Items	5-Year Survival	10-Year Survival	*P*-Value
Whole survival	92.7%	85.1%	
Age (Years)			
0–20	50%		0.017
21–40	95%	88.5%	
41–60	92.3%		
61–80	84.3%		
Gender			
Men	95.5%		0.55
Women	89.9%	85.0%	
Eye			
Right	92.9%	87.7%	0.58
Left	92.5%		
Visual Acuity (Decimal)			
<0.1	95.6%		0.50
0.1–0.49	94.1%		
0.5–079			
≥0.8	96.0%		
Intraocular Pressure (mm Hg)			
<10mmHg			0.44
10–21	94.9%		
>21mmHg			
Retinal Detachment			
Yes	97.2%		0.62
Tumor Position			
superior	95.5%		0.004
nasal	98.1%		
inferior	83.3%		
temporal	85.3%		
superior temporal	96.8%		
superior nasal	93.8%		
inferior nasal	91.1%		
inferior temporal	95.9%		
diffuse	53.3%		
Tumor Location			
ciliary body	97%		0.90
iris			
choroid	92.6%	84.2%	
Optic Disc Involvement			
no	92.6%	84.8%	
Tumor Size			
Small			<0.001
medium	96.5%		
large	83.9%		
Tumor Shape			
mushroom	95.2%		0.025
flat	83.3%		
hemispheric	98.2%		
irregular	87.7%		
Tumor Pathology			
spindle	91.0%		0.014
epitheloid	67.0%		
mixed	72.3%		
Treatment			
episcleral brachytherapy(EB)	98.4%		<0.001
local resection	94.8%	79.2%	
primary enucleation	84.8%		
EB + enucleation	85.5%		
Initial Treatment			
episcleral brachytherapy(EB)	95.5%		0.005
local resection	94.8%	79.2%	
primary enucleation	84.8%		

With adjusting for age, gender, tumor position, tumor size, tumor shape, tumor pathology and treatment type, the multivariate analysis revealed that only tumor size remained to be significantly (*P*<0.001; RR: 4.75; 95% confidence interval: 2.11, 10.7) associated with the overall survival (Table 3).

**Table 3:** Multivariate analysis by Cox Regression.

Items	Risk Ratio	95% Confidence Interval	*P*-Value
Age Group	1.18	0.65, 2.14	0.59
Tumor Pathology	1.57	0.78, 3.18	0.21
Tumor Size	4.75	2.11, 10.7	<0.001

## Discussion

Our retrospective hospital-based study with cumulative inclusion of 582 Chinese patients with uveal melanoma over a ten-year period revealed a 5-year survival rate of 92.7%. In univariate analysis, the higher overall survival rate was associated with younger age, location of the tumors in the nasal meridian, smaller tumor size, hemispheric tumor shape, histological tumor cell type (spindle cell type versus mixed cell type versus epitheloid cell type), and the type of treatment (episcleral brachytherapy versus local tumor resection and versus primary enucleation) (Table 2). In the multivariate analysis, only larger tumor size remained to be significantly associated with a less favorable overall survival (Table 3).

An overall survival rate at 5 years was of 92.7% (or a mortality related to uveal melanoma of 7.3%) in the Chinese patients of our study was higher than the 5-year survival rate found in previous studies in Western countries [6–12]. In a study from Spain, the mortality rate related to uveal melanoma at 5 years after diagnosis was of 11.6% [6]. Similar results were obtained in an investigation by Frenkel and colleagues with a 5-year mortality rate of 11.4% [7]. These figures are lower than those reported from the United States with a reported rate of 20% at 5 years [8]. Interestingly, these survival rates have remained mostly unchanged for the last 30 years [2]. Studies from Scandinavia and Great Britain showed a 5-year uveal melanoma related mortality of approximately 30% [9–11]. In a retrospective, nonrandomized, hospital-based interventional case series performed by Shields and colleagues on 8100 patients with uveal melanoma, the rate of metastasis, as assessed in multivariate analysis increased with older age (*P*<0.001), ciliary body location (*P*<0.001), increasing tumor base (*P*<0.001), increasing tumor thickness (*P*<0.001), tumor pigmentation (*P* = 0.001), subretinal fluid or retinal detachment (*P* = 0.001), intraocular hemorrhage (*P* = 0.045), and extraocular extension (*P* = 0.036) [12]. Compared with Caucasians, despite a relative risk for metastasis of 0.31 for African Americans, 0.73 for Hispanics, and 1.42 for Asians, there was no statistical difference in metastasis, or death from uveal melanoma based on race, so that the authors concluded that uveal melanoma showed similar prognosis for all races. The reasons for differences in the survival rate between the various studies have remained unclear yet. One of the reasons may be genetic differences between the study populations, in particular if the results of our study on Chinese patients are compared with the findings obtained in several investigations on Western populations.

In our study, the medium age at diagnosis of the uveal melanomas was 44 years. A similar age at the time of diagnosis was reported in a study on 103 patients from India with uveal melanoma [13]. This age was considerably younger than the age of the participants in the previous studies from other countries in which the uveal melanomas were diagnosed mostly in the sixth decade of life at a mean age of approximately 55 years [1,2,14]. In the univariate survival analysis, younger age was significantly associated with longer survival. When adjusted for other factors such as tumor size, location, and histological cell type, age was no longer significantly associated with survival. This finding was consistent with the results obtained in previous studies in which age at the time of diagnosis was not significantly correlated with the prognosis [15–17]. Other studies suggested that the survival was longer in children with uveal melanoma as compared with adults [18,19]. Kaliki and associates found that younger age at the time of diagnosis of uveal melanoma was associated with a lower rate of metastasis compared with middle-aged adults and elderly adults. The authors had stratified their study population into age groups (young [≤20 years], mid‑adults [21–60 years], older adults [>60 years]) and they had matched the age groups for clinical predictive factors for metastasis such as gender, tumor location, tumor basal diameter, tumor thickness, extraocular extension and follow‑up duration [20].

In our study, gender was not significantly associated with the survival rate, and both sexes had roughly the same proportion on the total study population. Correspondingly, the COMS group did not find significant differences in uveal melanoma-related metastasis and death between men and women [21]. In disagreement with our study, Zloto *et al*. found in a study of 723 patients with uveal melanoma that men had a worse prognosis with a higher rate of melanoma‑related metastasis and death than women had. In their study, melanoma‑related mortality in the first 10 years was two‑fold higher in men than in women [21]. In multivariable regression analysis, Rietschel and colleagues also detected that men had a significantly higher risk of melanoma‑related mortality than women had [22]. It was discussed that the lower metastatic rate in women could have been related to hormonal factors associated with estrogens [23].

Tumor size defined as the largest basal diameter and the largest height of the tumor has been one of the most important clinical prognostic features of uveal melanomas [24–26]. In our study, tumor size was the only factor which was significantly associated with the survival rate in the multivariate analysis. The 5-year survival rate was 96.5% in the group of patients with a medium-sized uveal melanoma, and it was 84.8% in patients with larger tumors. The number of small tumors was too small to be statistically analyzed. The reason for the low frequency of small uveal melanomas in our study population may have been the medical infrastructure in China. In particular in rural areas, regular health examinations are not common, and diseases may only rarely be detected in their early stages. The medium-sized tumor trial (2.5–10 mm tumor thickness and <16 mm basal diameter) by the COMS group revealed that the 5-year, 10-year and 12-year melanoma‑related mortality was 10%, 18%, and 21%, respectively, for patients in the iodine-125 brachytherapy treatment arm, and that it was 11%, 17%, and 17%, respectively, for the patients in the enucleation treatment arm [23]. In the large-tumor trial (>10 mm tumor thickness or >2 mm tumor thickness and >16 mm basal diameter) by the COMS group, the melanoma‑related mortality at 5 years and 10 years was 28% and 40%, respectively, for patients in the enucleation treatment arm and it was 26% and 45% for the patients receiving an external beam radiotherapy prior to enucleation [24,25]. In a long term study of 289 patients with uveal melanoma, Kujala *et al*. found a significant association between the largest basal diameter of the tumor and a higher melanoma‑related mortality [27]. In a study of 8,033 uveal melanoma patients by Shields *et al*. increasing tumor thickness was associated with an increasing risk for metastasis. Kaplan–Meier estimates of metastasis at 5 years, 10 years and 20 years was 6%, 12%, and 20% for small melanoma (<3 mm tumor thickness), 14%, 26%, and 37% for medium melanoma (3.1–8 mm), and 35%, 49%, and 67% for large melanoma (>8 mm), respectively. Each millimeter increase in tumor thickness was associated with approximately a 5% increase in the risk for metastasis at 10 years and a hazard ratio of 1.08 [26].

Tumor cell type has been considered to be an important prognostic factor for the survival of patients with uveal melanomas [28–33]. Callender initially proposed a classification system for uveal melanomas including the types of spindle A cells, spindle B cells, epitheloid cells, mixed cell type, and fascicular and necrotic cell types [30]. This classification was later modified to include spindle A cell type tumors, spindle B cell type tumors, epitheloid cell type tumors, and mixed cell type tumors. The modified Callender classification showed an improved correlation between the cell type and mortality [31]. In our study, we classified the cell type as spindle, epitheloid, and mixed, and the tumor cell type was associated with the survival rate in univariate analysis. In the multivariate analysis, the association lost its statistical significance. It is in contrast to a previous study by Paul and colleagues, who examined 2652 enucleated eyes with uveal melanoma and who found a 15‑year mortality for spindle A tumor of 19%, for spindle B tumors of 26%, for mixed spindle B and epitheloid tumors of 59%, and epitheloid tumors of 72% [32]. The 15‑year mortality of patients with melanomas of the mixed cell type was three times that of patients with tumors of the purely spindle cell type [28]. Previous studies reported that the spindle cell uveal melanomas had the best prognosis, mixed cell type melanomas an intermediate prognosis, and epitheloid cell type melanoma had the worst prognosis [28,29,32,33]. The prognosis decreased with an increasing number of epitheloid cells per high power field (HPF) in the microscopical examination. In a study of 232 enucleated eyes from patients with uveal melanoma, the 10‑year survival was 82% in patients with less than 0.5 epitheloid cells/ HPF, 55% for 0.5 to 4.9 epitheloid cells/HPF, and 33% in patients with >5 epitheloid cells/HPF [33].

Our results should be interpreted with some limitations in mind. First, the follow up period was relatively short for a major part of the study population. Second, we did not carry a molecular genetic examination of the tumor specimen. Recent studies have shown that the genetic pattern of the uveal melanoma is of high importance for the development of metastasis and survival [34–37]. Third, while in Caucasians many uveal melanomas are amelanotic or only lightly pigmented, the pigmentation of the uveal melanomas in our study could hardly be assessed since in Chinese as compared to Caucasians the melanin pigment is generally markedly more pronounced. Assessment of the tumor pigmentation may be of importance however, since previous studies showed that tumor pigmentation and melanogenesis can affect behavior and sensitivity of melanomas to chemotherapy, radiotherapy and immune therapy [38–40]. By the same token, and in contrast as found in our study, Shields and colleagues reported for their Asian group of patients, that 67% were pigmented, while 14% of the tumors were non-pigmented and 8% of the tumor showed a mixed degree pf pigmentation [12]. Future studies may address the reason for this difference in the pigmentation of uveal melanomas between Asians examined in the USA and most of them living in the USA, and Chinese living and examined in mainland China.

In conclusion, in this first study on clinical characteristics of uveal melanomas of a relatively large group of patients from China, the onset age was considerably younger and survival rate better than in studies from Western countries. Tumor size was most significant factor for survival.
